# The structure, processes, and outcomes of stroke rehabilitation in Ghana: A study protocol

**DOI:** 10.3389/fneur.2022.947289

**Published:** 2022-08-24

**Authors:** Cosmos Yarfi, Gifty Gyamah Nyante, Anthea Rhoda

**Affiliations:** ^1^Department of Physiotherapy, Faculty of Community and Health Sciences, University of the Western Cape, Cape Town, South Africa; ^2^Department of Physiotherapy and Rehabilitation Sciences, University of Health and Allied Sciences, Ho, Ghana; ^3^Department of Physiotherapy, School of Biomedical and Allied Health Sciences, University of Ghana, Accra, Ghana

**Keywords:** stroke, outcomes, conventional, complementary, rehabilitation, structure, processes and Ghana

## Abstract

**Background:**

Conventional and complementary treatments are often used in rehabilitation for persons with stroke. The conventional treatment makes use of medications, physiotherapy, occupational, speech, and diet therapies, while the complementary treatment makes use of homeopathy, naturopathy, massage, and acupuncture. The structure, process, and outcomes of stroke rehabilitation using conventional or complementary treatments have not been empirically investigated in Ghana.

**Aims:**

This study aims to investigate the structure, process, and outcomes of stroke rehabilitation at the Korle Bu Teaching Hospital (KBTH) in Accra and Kwayisi Christian Herbal Clinic (KCHC) in Nankese-Ayisaa, Ghana, and to explore the experiences of persons with stroke.

**Methods:**

This study involves a mixed methods approach. This study will utilize three study designs, namely, cross-sectional, hospital-based cohort, and qualitative exploratory study designs. The objectives of the study will be achieved using three phases, namely, phase one will recruit health professionals and gather information on the structure and process of stroke rehabilitation at a conventional and complementary hospital using adapted questionnaires; phase two will determine the outcomes of stroke patients attending a conventional and complementary hospital facility at baseline, 2-, 3-, and 6-month follow-up using outcome measures based on the International Classification of Functioning, Disability and Health (ICF) model; and phase three will explore the experiences of stroke patients who use complementary or conventional treatment using an interview guide.

**Data analysis:**

IBM SPSS Statistics Version 27 will be used to analyze the data using descriptive and inferential statistics. Repeated measures of ANOVA will be used to determine the differences between variables at baseline, 2-, 3-, and 6-month post-stroke. The qualitative data will be transcribed and entered into Atlas Ti version 9.0. The data will be coded and analyzed using thematic areas that will be generated from the codes.

**Conclusion:**

The study protocol will provide a comprehensive overview of the structure, process, and outcomes of stroke rehabilitation in Ghana, incorporating both conventional and complementary treatment and rehabilitation into the stroke recovery journey. It will also inform clinical practice, with new insights on the experiences of stroke patients based on their choice of rehabilitation pathway.

## Introduction

Stroke rehabilitation is aimed at reducing the disability-related impact of stroke on individuals, enabling them to achieve independence, social integration, a better quality of life, and self-actualization (1). The importance of rehabilitation medicine in the attainment of optimal functioning after an injury or disease cannot be overemphasized (2). According to the World Health Organization, rehabilitation aims to enable persons with functional limitations to improve and maintain their optimal functional levels through the provision of tools to help attain independence (3).

Conventional medicine involves healthcare practices through which healthcare professionals treat symptoms and diseases in a medically supervised setting using therapy, drugs, radiation, and/or surgery (4). Complementary medicine involves healthcare practices that are not part of a country's conventional health practices and are not fully integrated into the dominant healthcare system, but are used along with mainstream healthcare (4, 5). Complementary medicine involves a health system that uses non-mainstream approaches together with conventional medicine in healthcare practice (4, 5). Conventional and complementary medicine treatments are often used in rehabilitation for stroke patients (6–10). The usage of complementary medicine and rehabilitation has a high prevalence in the treatment of stroke in Korea (54%) and India (67%) (11), as well as in developed countries such as the United States, Australia, France, and Canada, with its usage ranging from 42 to 70% (12). In Africa, complementary medicine and rehabilitation is widely used in most countries, and sometimes, it is the only source of primary healthcare (13–15); about 70% of the population in Ghana utilize complementary medicine and rehabilitation in one way or the other (16). Complementary medicine refers to a broad set of healthcare practices that are not part of the mainstream medical care in a country and are not fully integrated into the dominant healthcare system (17–19). It involves the use of non-mainstream approaches of healthcare such as chiropractic, acupuncture, homeopathy, herbal therapy, dietary, and psychological interventions (20, 21) together with conventional medicine (7, 22). Conventional medicine is the use of evidence-based treatments that are safe and effective, with rigorously tested procedures used as clinical practice guidelines (23). The focus of conventional medicine is more often on the treatment of existing ailments within the context of a specific scientific framework. Rehabilitation is a major part of conventional medicine treatment, forming a major part of patient care (24).

The structure, process, and outcomes (SPO) framework initially developed to assess the quality of healthcare has recently been used to examine outcomes related to differences in structure and process of rehabilitation and their association with outcomes post-stroke (8, 25, 26). The SPO framework will be utilized in this study in addition to the International Classification of Function, Disability and Health (27) to conceptualize outcomes post-stroke which include impairments, activity limitations, and participation restrictions of stroke patients utilizing conventional or complementary medicine and rehabilitation in Ghana.

By undergoing a specific stroke rehabilitation pathway, stroke patients can achieve their best possible functional independence, which ultimately improves their quality of life (7). Good outcomes after stroke have been seen in patients undergoing rehabilitation in the conventional setting (9, 28–32). Stroke outcomes have also been shown to be better in cases managed at the stroke units with multidisciplinary care (33). In the Ghanaian context, the coordination of stroke care after discharge from acute care is fragmented (34), with further rehabilitation after acute care poorly addressed due to accessibility of care issues such as inadequate medical facilities and financial constraints (34, 35). For this reason, some patients and/or their caregivers choose to use rehabilitation in a complementary or conventional setting as a treatment for stroke based on availability in the community and acceptability to their health beliefs or religious faith (7, 36), and sometimes as a substitute for the absence of conventional rehabilitation (7, 37). However, there is a paucity of information on the outcomes of stroke patients who use complementary medicine and rehabilitation in Ghana. In an era where complementary healthcare services are being introduced gradually to mainstream healthcare (16, 38, 39), it is important to investigate and document the structure, processes, and outcomes of complementary medicine and rehabilitation in a resource-constraint environment (40). At present, there are few studies on the outcomes of stroke patients who use conventional medicine and rehabilitation (41) and none on complementary medicine and rehabilitation in Ghana. Published literature has focused more on conventional medicine and rehabilitation, with less or no literature existing on complementary medicine and rehabilitation. This study will fill the gap by providing information on structure, process, and outcomes of stroke rehabilitation in a conventional and complementary hospital setting in Accra and Nankese-Ayisaa, Ghana.

The purpose of this study is to comprehensively describe the structure and processes of stroke rehabilitation to investigate the outcomes of stroke patients who utilize conventional or complementary medicine and rehabilitation. It is hoped that the findings can pave the way for more studies assessing the association between structure, process and outcomes of stroke rehabilitation in low-resource settings.

## Objectives

The study will be guided by the following objectives:

1. To determine the structure of stroke rehabilitation using conventional and complementary medicine at KBTH in Accra and KCHC in Nankese-Ayisaa, Ghana.
2. To determine the processes of stroke rehabilitation using conventional and complementary medicine at KBTH in Accra and KCHC in Nankese-Ayisaa, Ghana.
3. To determine the outcomes of participants in relation to their impairments, activity limitations, and participation restrictions at baseline, 2-, 3-, and 6-month follow-up at KBTH in Accra and KCHC in Nankese-Ayisaa, Ghana.
4. To explore the experiences of stroke patients about the process and outcomes of their rehabilitation at a conventional or complementary rehabilitation after 6 months of rehabilitation at KBTH in Accra and KCHC in Nankese-Ayisaa, Ghana.

## Methods

### Study design

This study will adopt a triangulation, mixed-method approach with quantitative and qualitative methods (42). The quantitative part will involve a cross-sectional and hospital-based cohort study of health professionals and stroke patients, respectively, while the qualitative part of the study will be a descriptive exploratory study of stroke patients in a conventional and complementary hospital in Accra and Nankese-Ayisaa, Ghana. The study will be carried out in three phases. The data will be collected at the stroke unit and physiotherapy department, KBTH, and the outpatient department and physiotherapy department, KCHC. A hospital-based cross-sectional study will be conducted from November 2021 to April 2022 on health professionals to gather information about their numbers and availability of equipment for rehabilitation at the hospital and the compliance with the agency for healthcare policy and research (AHCPR) using adapted questionnaires. The cohort study will assess outcomes of stroke patients using outcome measures at baseline, 2-, 3-, and 6-month follow-up from December 2021 to April 2023. The baseline measurement will be within a month after the start of stroke treatment. The qualitative exploratory design will be used to explore the experiences of stroke patients regarding their rehabilitation process and outcomes in a conventional or complementary rehabilitation setting from June 2022 to August 2022.

### Participants and setting

The study population will comprise all health professionals involved in stroke care at the study sites and all stroke patients at KBTH and KCHC during the study period fulfilling the inclusion criteria.

### Inclusion criteria for health professional participants

Eligible participants should:

i. be health professionals at the study sites,
ii. be involved in stroke rehabilitation, and
iii. consent to participate in the study.

### Exclusion criteria for health professional participants

Participants will be excluded from the study if they:

i. are students,
ii. are intern professionals, and
iii. do not consent to participate in the study.

### Inclusion criteria for stroke patient participants

Eligible participants should:

i. be aged 18 years and above,
ii. be clinically diagnosed with stroke using clinical signs and CT scan or MRI confirmation,
iii. be those within a month after starting rehabilitation,
iv. have a modified Rankin score (MRS) of <4,
v. willing to come for follow-up assessment at 2-, 3-, and 6-month post-recruitment into the study, and
vi. speak either English, Ga or Twi (Ghanaian local languages).

### Exclusion criteria for stroke patient participants

Participants will be excluded from the study if they:

i. have other neurological conditions such as previous head injury or spinal cord injuries, dementia, and seizures;
ii. have psychological or mental instability;
iii. have the inability to communicate verbally and comprehensively as a result of global aphasia;
iv. have stroke-like symptoms due to subdural hematoma, brain tumor, encephalitis, or head trauma; and
v. the patient or family do not provide informed consent.

Phase one of the study will be carried out at the stroke unit and physiotherapy department, KBTH and the out-patient department and physiotherapy department, KCHC; phases two and three will be carried out at the physiotherapy department of the two facilities. The KBTH is the premier and largest teaching hospital, located in the Greater Accra region of Ghana, with a population of 5.4 million (43). The Accra metropolis, in the Greater Accra region, has a population of 1,665,086 spread across 60 square kilometers, consisting of both urban and peri-urban areas (44). The KBTH is a 2,000-bed capacity referral hospital in the southern part of Ghana, with a stroke unit incorporating multidisciplinary professionals for stroke management (45).

The KCHC is a herbal clinic located in Nankese-Ayisaa, in the Eastern region of Ghana, which has a population of 2.9 million (43). Nankese-Ayisaa is part of the Suhum Municipality, ~60 km from Accra, the national capital. Suhum Municipality has a population of 90,358 spread across 359 square kilometers, consisting of both urban and rural areas (44). The hospital uses complementary medicine (herbal medicine) and nutritional supplements in the treatment of stroke patients. They also undertake outpatient rehabilitation services using massage therapy, herbs, exercise therapy, and dietary counseling. The facility focuses on stroke care using herbal preparations and food supplements under the standards set by the Traditional Medical Practice Council in Ghana (16).

### Recruitment

All health professionals involved in stroke care at the study sites who sign the consent form will be recruited for the study. The estimated average monthly population of new stroke patients at the physiotherapy department of KBTH and KCHC is 20 and 10, respectively, during the 2021 mid-year performance review (46, 47). A power calculation at 80% power, 5% level of significance, and 95% confidence interval was used to determine the number of participants to be recruited. Assuming a 70 and 50% recovery rate for patients using conventional and complementary treatment, respectively, a 10% non-response rate and with standard approximations for loss to follow-up, a sample size of 200 is estimated. Therefore, a total of 100 participants will be recruited at each study site to retain statistical power. All eligible participants will be consecutively enrolled in the study. The study will be carried out over a window period of 18 months.

A sample size of up to 20 participants will be selected purposively, with 10 at each study site for the qualitative study. “Information power” meaning the amount of relevant information needed for the study will determine the number of participants for the study (48). The selection will be done based on the age and gender of the participants.

### Data collection

The study will adhere to the ethical guidelines of the Declaration of Helsinki in 2013 (World Medical Association Declaration of Helsinki) (49). All study participants will be informed about the purpose and objectives of the study and asked to sign an informed consent form prior to participation. The right of participants to safeguard their anonymity and integrity will be respected. All participants will be adequately informed of the aims, methods, consent to participation, potential risk/benefits, voluntary participation, privacy/confidentiality, compensation, declaration of conflict of interest. Signing the informed consent is necessary for recruitment.

#### Data collection instruments

The data collection will involve three phases, namely, structure and process of care, outcomes post-stroke, and experiences with rehabilitation process and outcomes. The questionnaires for the quantitative study will be validated and tested for reliability by administering them to health professionals at the physiotherapy department of Komfo Anokye Teaching Hospital (KATH) and the outpatient department of Amen Scientific Herbal Hospital (ASHH) in Kumasi in a pilot study. The interview guide for the qualitative study will also be piloted on stroke patients at KATH and ASHH to determine whether it answers the research questions for the study and the time it will take to administer the interviews. The KATH and ASHH have similar characteristics as the study sites for the study in terms of structure and population required for the study.

#### Phase 1: Structure and process of care

An adapted questionnaire redesigned by Rhoda (8) based on the taxonomy developed by Hoenig et al. (50) will be used to collect data on the structure of stroke rehabilitation. The questionnaire consists of four main domains. Domain one consists of socio-demographic information of professionals such as age, gender, highest qualification, year of qualification; domain two consists of information on professional expertise such as the availability of professionals and the interventions used; domain three consists of capacity building of professionals such as attendance of continuous professional education, presence of team meetings, and use of outcome measures; and domain four consists of rehabilitation equipment available at the facility.

An adapted questionnaire will be used to measure the process of care for stroke patients by compliance with the agency for healthcare policy and research (AHCPR) and clinical guidelines for post-stroke rehabilitation (26). The questionnaire uses a Likert scale to gather information relating to multidisciplinary team coordination, baseline assessment of patients, monitoring and evaluating progress, and management of impairments and functional limitations of patients.

#### Phase 2: Outcomes of rehabilitation

Table 1 shows the outcome measures, their assessment methods, and assessment time points. Primary and secondary outcome measures will be assessed in each participant at different time points (baseline, 2-, 3-, and 6-month post-stroke). The primary outcome will be the improvement of voluntary movement of the limbs and basic mobility. The secondary outcome measures will be improvements in stroke-specific functional and quality-of-life measures based on the impairments, activity limitations, and participation restrictions: Montreal Cognitive Assessment Scale (MoCA), Stroke Rehabilitation Assessment Movement (STREAM), Time Up and Go (TUG) test, 10-meter walk (10 MW) test, Tinetti Scale (TS), Reintegration to Normal Living Index (RNLI), and Health Related Quality of Life for Stroke Patients (HRQOLSP). The instruments have been used in sub-Saharan Africa with good reliability measures (51–57).

**Table 1:** Summary of outcome measures and respective methodology.

**Outcome**	**Measurement method**	**Time points (months)**
Improvement of voluntary movement of the limbs and basic mobility	STREAM	Baseline, 2-, 3- and 6-months post-stroke
Gait and balance	TS test	Baseline, 2-, 3- and 6-months post-stroke
Walking ability	TUG test	Baseline, 2-, 3- and 6-months post-stroke
Cognitive function	MoCA test	Baseline, 2-, 3- and 6-months post-stroke
Walking speed	10 MW test	Baseline, 2-, 3- and 6-months post-stroke
Reintegration in terms of ADLs, social and recreational activities and interactions with others	RNLI	Baseline, 2-, 3- and 6-months post-stroke
Quality of life	HRQOLISP	Baseline, 2-, 3- and 6-months post-stroke

#### Phase 3: Experiences of participants

An interview guide will be developed by the authors from the literature. The guide will gather information on the experiences of stroke patients on the process and outcomes of their rehabilitation.

### Data collection procedure

#### Phase 1: Structure and process of care

The study will commence after permission from authorities at the study sites has been granted and the necessary ethical clearances are given. Standardized training in all aspects of the study instruments will be provided to research assistants for 1 week on how to administer the instruments and score the participants. The first author will attend one of the clinical meetings for health professionals at the study sites. The purpose and objective of the study will be explained to them, and they will be invited to participate in the study by completing the questionnaires after signing a consent form. The adapted questionnaires will be administered to the health professionals after the clinical meeting and follow-up meeting with them. Weekly reminders and periodic visits will be conducted at the sites to increase the response rate of the questionnaires. An online generated version of the questionnaires will also be sent for participants who prefer that. The questionnaires will take about 15 min to complete and are available in English.

#### Phase 2: Outcomes of rehabilitation

The records unit of the study sites will be approached for the list of stroke patients and the contacts of those receiving care or attending physiotherapy at the facility. The patients will be contacted and the purpose of the study will be explained to them and their caregivers. An appointment will be set up for screening, and participants meeting the inclusion criteria are invited to participate in the study. Those who agree will be recruited into the study, after signing a consent. In addition, the first author will approach patients who report for physiotherapy services for recruitment into the study. The research assistants will administer the bio-demographic questionnaire first, followed by the stroke levity scale, to assess the severity of stroke (41), then the other self-reported questionnaires such as RNLI and HRQOLSP at baseline, and finally the observer-rated questionnaires such as STREAM, TS, and MoCA. Once the observer-rated instruments are completed, the first author will continue to conduct objective assessments such as the TUG and 10 MW tests.

The TUG will be performed with patients seated on a chair with arm rest and a measured distance of 3 m from the chair. The time taken for the patient to stand up from the chair and walk toward the 3-m mark and turn back and sit on the chair will be recorded. The participant may use the arms of the chair to stand up or sit down and walk as fast and safe as possible. The participant can wear their regular footwear, may use any gait aid or assistive device that they normally use during ambulation, but may not be assisted by another person. There is no time limit. They may stop and rest (but not sit down) if they need to. The time for two trials will be recorded.

The 10 MW test is performed by recording the time patients walk without assistance for a distance of 10 m. The time is recorded for intermediate 6 m to allow for 2 m of acceleration and deceleration. The use of assistive devices or physical assistance is allowed, but should be kept consistent and be documented. The documentation will include normal and fast walking speed. The time for three trials will be recorded, and the average will be calculated for normal and fast speed. Once the researcher and assistants have finished collecting the baseline data, the participants will be informed that they would be contacted for an appointment for the 2-, 3-, and 6-month follow-up assessments. The follow-up assessments will be done by the same researcher or assistant who had conducted the baseline assessments within a window period of 7 working days either before or after the actual date. The estimated duration for all the assessments will be 1 h. The questionnaires will be available in either English, Twi or Ga and will be administered in the language the participant prefers.

#### Phase 3: Experiences of participants

The first author will advertise phase 3 of the study to eligible participants who have completed 6 months of stroke rehabilitation at either the conventional or the complementary facility. The purpose and objectives of the study will be explained to the participants. Participants who agree to be part of the study will have either a written consent or an audio consent taken; permission will also be taken to record the interviews. The first author will administer a one-on-one audio interview with participants using an interview guide in a quiet venue, chosen by the participant, either in their homes or at the clinic. The first author will take notes during the interview, which will last for a maximum of 40 min. The interview guide will be available in either English, Ga or Twi and will be administered in the language the participant prefers.

### Translation of the questionnaires

The outcome measures are available in English and will be translated from English to Twi and Ga (Ghanaian local languages) by a team of translators, with experience in questionnaire translation from the University of Ghana and Kwame Nkrumah University of Science and Technology. The translated questionnaires will be back translated into the original language (English) by another translator. All the translators will not be associated with the study, and the back translators will be independent of the first translators. Modifications and changes will be done taking into account the local context where needed (58) after agreement with the translators. The content of the translated items will be checked to see if it remained the same irrespective of the translation process.

### Data analysis

#### Quantitative data

The data will be captured and stored in an encrypted Microsoft Excel file. Following data collection, the data will be cleaned and checked for accuracy. The data will be transferred into Statistical Package for the Social Sciences (SPSS) version 27 and analyzed using both descriptive and inferential statistics. Frequencies of socio-demographic and outcome variables will be determined. The frequencies will relate to data collected at baseline, 2-, 3-, and 6-month post-stroke. The frequencies will be presented in the form of means and standard deviations or medians and interquartile ranges depending on the distribution of the dataset. The Kolmogorov–Smirnov test will be used to assess the normality of the data. For participants who are unable to perform any of the items on the scales at baseline and at follow-up visits, a value of 0 will be assigned to them, because this score correctly reflects the subjects' inability to perform any of the items. A repeated measure ANOVA will be conducted on all outcome measures with time (pre-post) as within-subject variables and between-subject variables at each study sites. Comparisons will be made with the entire sample as well within each subgroup classified by scores on the stroke levity scale (mild, moderate, severe). Comparisons among all of the measures will be made for the 4-time intervals, namely, baseline, 2-, 3-, and 6-month assessments. An independent *t*-test will be used to compare the differences in age, gender, improvement of voluntary movement of the limbs, basic mobility, gait and balance, walking ability, cognitive function, walking speed, quality of life, and reintegration (activities of daily living, social, recreational activities, and interactions with others). To compare the changes at each site while adjusting the effect of confounding variables, the covariance analysis model or the relative change analysis will be used. Questionnaire scores between baseline, 2-, 3-, and 6-month post-stroke assessments will be compared using a generalized linear model. In all cases where differences occurred between baseline, 2-, 3-, and 6-month post-stroke assessments, *post-hoc* analyses with Bonferroni's adjustments for multiple comparisons will be done. For missing data imputation, the last value carried forward (LVCF) method will be used. A *p*-value of <0.05 will be considered statistically significant.

#### Qualitative data

The data collected will be analyzed using Atlas Ti version 9.0. The interviews in Ga and Twi will be translated into English and checked by an independent translator to make sure all the interviews are correctly translated until consensus in the final translation is agreed on. The interviews together with the translated one and the field notes will be transcribed into text (English). The text will be entered into Atlas Ti version 9.0 and analyzed using thematic analysis. The analysis will be performed by reading the text for familiarization with the data, establishing meaningful patterns, generating initial codes, searching for themes among the generated codes, reviewing the themes, defining and naming the themes, and producing the final report. Data from field notes will also be used for the analysis to enhance the results. The themes and sub-themes that will emerge from the data will be supported with verbatim quotes from the participants' transcribed data.

#### Trustworthiness of qualitative data

The credibility of the research will be ensured by explaining to the participants that participation in this study is completely voluntary, and they can choose not to participate in this research. The researcher will gather the required information from the participants during the interview process using additional probes. The credibility of the research will also be ensured by keeping a reflexive journal where the researcher's assumptions, thoughts, and ideas about the Research Topic and the disclosures of the participants during the interview process will be noted. Member validation will be done to ensure that the participants' experiences about stroke rehabilitation will be accurately represented in the data gathered. The transcribed data will be given to the participants to review during the data analysis and to provide feedback to ensure that their transcribed interviews were accurately recorded and the themes generated are meaningful to them. Transferability and dependability of the study will be ensured by giving detailed information about this study, as documented in the comprehensive methodology, and by keeping field notes using a reflexive journal. Lastly, conformability will be obtained through an audit trail of the procedures done.

## Discussion

This study protocol offers an investigation of the structure, process, and outcomes of stroke patients in two different contexts 6 months post-stroke The research questions that this study aims to address are as follows:

1. The structure, processes, and outcomes of care for stroke patients using either conventional or complementary rehabilitation in Accra and Nankese-Ayisaa, Ghana.
2. The experiences of stroke patients about the process and outcomes of their rehabilitation at a conventional or complementary facility after 6 months of rehabilitation in Accra and Nankese-Ayisaa, Ghana.

Ghana has a vibrant pluralistic healthcare system made up of both mainstream biomedical (conventional) and complementary (herbal) health systems, which are all involved in stroke care and rehabilitation (59). In many rural and semi-urban areas in Ghana, most patients with stroke tend to use complementary medicine treatment either exclusively or in parallel with conventional medicine (40, 60) for varied reasons ranging from faith and cultural congruence to accessibility, cost, and belief that these approaches are safe (40, 61, 62).

Some stroke patients and their family members believe stroke is a spiritual illness caused by evil spirits or witches and as such the need to resort to herbal and faith healing clinics after discharge from conventional hospitals (36, 40, 62–64). Some of these reasons have led to the patronage of complementary rehabilitation among stroke patients in addition to conventional rehabilitation in Ghana (40, 62). In an era where complementary healthcare services are being introduced gradually to mainstream healthcare (16, 38), it is important to investigate and document the structure, processes, and outcomes of both conventional and complementary medicine treatment in a resource-constraint environment (40).

The study will provide information on the structure and process of stroke rehabilitation in a conventional and a complementary setting and explore the experiences of stroke patients who attend rehabilitation in two different settings. The study will also determine the outcomes of stroke rehabilitation in a conventional and complementary rehabilitation setting in Ghana after a 6-month follow-up. This study will inform clinical practice for stroke rehabilitation in Ghana and may improve stroke management.

In conclusion, the results will provide a comprehensive overview and insight into stroke rehabilitation in Ghana in terms of structure and process and outcomes of care, incorporating both conventional and complementary treatment and rehabilitation into stroke survivors' recovery journey. It will bring out the clinical and research implications of the different pathways of the current overview of the structure, process, and outcomes of stroke rehabilitation in Ghana by gathering data from both health professionals and persons with stroke.

## Ethics statement

The studies involving human participants were reviewed and approved by Biomedical Sciences Research Ethics Committee of the University of the Western Cape, South Africa (BM20/5/25), the University of Health and Allied Sciences Research Ethics Committee (UHAS-REC A.1 [15] 20-21) and the Scientific and Technical Committee/Institutional Review Board of Korle Bu Teaching Hospital, Ghana (STC/IRB/000113/2020). The patients/participants provided their written informed consent to participate in this study.

## Author contributions

CY and AR conceptualized the study with input from GGN. CY drafted the manuscript and first version of the manuscript with input provided by GGN and AR. The draft manuscript was reviewed by AR and GGN. AR assisted with letters for partial funding for the data collection. CY drafted the ethical clearance documents for the study with assistance from AR. Study design and methods were conceptualized by CY, GGN, and AR. CY addressed all co-authors' comments and made the final draft of the manuscript. All authors reviewed the draft manuscript and provided approval for the final manuscript.

## Funding

The study received funding from the Ghana Education Trust Fund (GetFund) and Institute of Health Research Faculty Development Grant, University of Health and Allied Sciences, Ho, Ghana.

## Conflict of interest

The authors declare that the research was conducted in the absence of any commercial or financial relationships that could be construed as a potential conflict of interest.

## Publisher's note

All claims expressed in this article are solely those of the authors and do not necessarily represent those of their affiliated organizations, or those of the publisher, the editors and the reviewers. Any product that may be evaluated in this article, or claim that may be made by its manufacturer, is not guaranteed or endorsed by the publisher.
