# Supplementary material for: The structure, processes, and outcomes of stroke rehabilitation in Ghana: A study protocol
Source: Front Neurol. 2022 Aug 24;13:947289. doi: 10.3389/fneur.2022.947289 (PMC9449840; doi:10.3389/fneur.2022.947289)
Supplement: Supplementary file 1 [file Data_Sheet_1.docx]

**APPENDIX I**

**STRUCTURE OF CARE**

**Section A: Socio-Demographic Information of Professional**

1. Participant code: _______________
2. Facility working: _____________________________
3. Date of completing this form: (dd/mm/yyyy): ___________________
4. Address: _______________________________
5. Contact: _______________________________
6. Age (in years): __________________________
7. Gender: [ 1 ] Male [ 2 ] Female [3] Other/please specify______________
8. Marital status: [ 1 ] Single [ 2 ] Married [ 3 ] Divorced [ 4 ] Separated

[ 5 ] Cohabitating [ 6 ] Others/please state ____________________________

1. What is your profession? [ 1 ] Physiotherapist [ 2 ] Occupational therapist

[ 3 ] Speech therapist [ 4 ] Acupuncturist [ 5 ] Naturopathic doctor

[ 6 ] Herbal doctor [ 7 ] Other/please name ____________________

1. Your highest professional qualification: [ 1 ] Certificate [ 2 ] Diploma [ 3 ] BSc

[ 4 ] Postgraduate [ 5 ] Other/please specify ________________

1. Year of qualification? ___________________
2. Total number of working experience in years: ___________ years
3. How many years of experience do you have treating **stroke patients?** ______________
4. Do you have a **post basic training** after your entry qualification?

[ 1 ] Yes [ 2 ] No

1. If you answered **Yes in question 14**, please indicate the post basic training you did? ___________________________________________
2. Tick all the rehabilitation services received by stroke survivors at this facility.

[ 1 ] Physiotherapy [ 2 ] Occupational therapy

[ 3 ] Speech therapy [ 4 ] Acupuncture [ 5 ] Naturopathy

[ 6 ] Herbal therapy [ 7 ] Diet therapy [8] Psychology

[9] Other/please name _____________________________________________________

**SECTION B. INFORMATION ON PROFESSIONAL EXPERTISE**

1. **Please indicate the available health professionals in this facility by ticking (√ ) and write the number the total number in the last column.**

| **Health Professionals** | **Available [**$\boldsymbol{\surd}$**]** | **Not-available [**$\boldsymbol{\surd}$**]** | **Total number** |
| --- | --- | --- | --- |
| Physiotherapists |  |  |  |
| Occupational therapists |  |  |  |
| Dieticians |  |  |  |
| Medical Officers |  |  |  |
| Pharmacist |  |  |  |
| Nurses, |  |  |  |
| Social workers |  |  |  |
| Psychologists |  |  |  |
| Speech therapists |  |  |  |
| Physiotherapy Technicians/Assistants |  |  |  |
| Homeopathic doctors |  |  |  |
| Acupuncturists |  |  |  |
| Herbal doctors |  |  |  |
| Physiotherapy interns |  |  |  |
| Students/Specify |  |  |  |
| Others/please specify |  |  |  |

1. Within the past 3 months, have you used any orthotic or supporting device to treat stroke patients? [ 1 ] Yes [ 2 ] No (**If you answered No, move to question 18**)
2. **If You answered YES in question 2, name the type of equipment/supporting device you used**:_______________________________________________________________
3. Indicate below whether the activities/interventions mentioned are mostly used, moderately used or not used at all using **the numbers attached to them.**

**1–Mostly used 2–Moderately used 3–Not used at all**

| **SN** | **INTERVENTIONS** | **FREQ OF USE [indicate using 1, 2 or 3]** |
| --- | --- | --- |
| 1. | **Mobilization, manual joint mobilization, stretching & palpation:** (including pain assessment) passive relation and massage e.g passive or self-assisted movements, weight bearing on upper/lower limbs. |  |
| 2. | **Reeducating selective movements:**  Movement coordination exercises, strengthening exercises, bridging, moving knees from side to side. |  |
| 3. | **Lying and lying position:**  Positioning and correcting alignment. |  |
| 4. | **Sitting and sitting balance:**  Positioning, weight shifting, equilibrium reactions. |  |
| 5. | **Standing and standing balance:**  Positioning, weight shifting, equilibrium reactions, walking on spot, stepping on and off steps, practicing swing phase of walking from standing position. |  |
| 6. | **Sensory and visual perception training and cognition:**  Treating neglect, hemianopia, neurophysiological training (memory, language, orientation, space, body, gestures and planning) |  |
| 7. | **Transfers:** rolling, sitting to lying and vice versa, sitting to standing, moving from the floor, moving into and out of positions. |  |
| 8. | **Ambulatory activities:** wheelchair activities, walking with or without assistance, stair climbing with or without assistance. |  |
| 9. | **Personal Activities of Daily Living:** washing**,** dressing, toileting, shaving, feeding, preparing food, brushing teeth, combing hair. |  |
| 10. | **Domestic** **Activities of Daily Living:**  Cooking, cleaning dishes, laundry, ironing, tidying house, others |  |
| 11. | **Leisure and social activities:** such as visiting family and friends, playing ludo, ‘oware’ |  |
| 12. | **Work-related activities:** eg going for shopping/market, using the computer to work etc |  |
| 13. | **Using augmentative or alternative communication devices or aids** |  |
| 14. | **Tongue strengthening exercises** |  |
| 15. | **Verbal expressions, object matching, naming objects** |  |
| 16. | **Auditory comprehension, perceptual skills** |  |
| 17. | **Facial strengthening exercises** |  |
| 18. | **Reassurance and counseling of patients and relatives** |  |
| 19. | **Education of patients/relatives about their condition** |  |
| 20. | **Hand eye coordination exercises** |  |
| 21 | **Acupuncture:** Using fine needles to insert in the skin to treatment physical and mental conditions at meridians, or pressure points. |  |
| 22 | **Herbal medicines:** using traditional medicinal plants |  |
| 23 | **Homeopathy:** Using minute doses of natural substances as treatment. |  |
| 24 | **Light therapy:** using infra-red or other forms of light therapy |  |
| 25 | **Exercise therapy:** plan of physical activities designed and prescribed to facilitate the patients to recover from diseases and any conditions, which disturb their movement and activity of daily life or maintain a state of well‐being. |  |
| 26 | **Dietary intervention:** resolving or improving client’s condition by provision of advice, education, or delivery of the food component of a specific diet or meal plan tailored to the patient needs. |  |
| 27 | **Massage:** manipulation of the body's soft tissues using a device/machine. |  |
| 28 | **Manual therapy:** Manual traction, massage techniques using the hands, fingers, elbows, knees, forearms, feet, mobilization/manipulation of limbs, passive range of motion of joints. |  |
| 29 | **Tai chi:** Combining deep breathing and relaxation with flowing movements. |  |
| 30 | **Yoga:** physical, mental, and spiritual exercises for stretching and strengthening. |  |
| 31 | **Music therapy:** using music as a treatment or playing music while carrying out treatment |  |
| 32 | **Miscellaneous techniques** (chest physiotherapy) |  |
| 33 | **Miscellaneous techniques** (hydrotherapy) |  |
| 34 | **Miscellaneous techniques** (electrotherapy) |  |
| 35 | **Others/ Please specify any additional activity you teach your patients** |  |

**SECTION C: CAPACITY BUILDING OF PROFESSIONALS**

1. Have you attended any continuing professional education, courses, workshops, seminars/lectures on stroke rehabilitation after your last qualification/degree?

[ 1 ] Yes [ 2 ] No

| **Course name** | **Year attended** | **No of days (If full day)** | **Half day** | **No of hours (If Half day)** |
| --- | --- | --- | --- | --- |
|  |  |  |  |  |
|  |  |  |  |  |
|  |  |  |  |  |
|  |  |  |  |  |
|  |  |  |  |  |
|  |  |  |  |  |

1. If you answered **Yes in question 1,** indicate/ please write the names of the courses or workshops you have attend.
2. Do you have team meetings in your clinic/department to discuss management of stroke patients? [ 1 ] Yes [ 2 ] No
3. **If Yes, state frequency of meeting**. [ 1 ] once a week [ 2 ] once in two week s

[ 3 ] once in a month [ 4 ] others/specify _______________

1. Who attends the meetings at the department? Indicate **Tick (√)** all the professionals who attends the meetings.

| **Professionals** | **Tick (√)** |
| --- | --- |
| Physiotherapists |  |
| Occupational therapists |  |
| Dieticians |  |
| Social workers |  |
| Medical Officers |  |
| Pharmacists |  |
| Nurses |  |
| Speech therapists |  |
| Physiotherapy Technicians/Assistants |  |
| Physiotherapy Interns |  |
| Homeopathic doctors |  |
| Students |  |
| Others/please specify |  |

1. On average how long does a stroke treatment session lasts (everyday) per patient at the clinic? (in **minutes) ___________________**
2. How many stroke patients do you usually assess and treat in a week on average? _________________
3. How often do you treat stroke patients at the clinic on average? [ 1 ] everyday

[ 2 ] once in a week [3] twice in a week [ 4 ] once in two weeks

[ 5 ] once in a month [ 6 ] other/please specify __________________________

1. Please indicate the treatment approach you have for patients with stroke? [1] individuals

[ 2 ] group therapy [ 3 ] home visits [ 4 ] other/specify_________________

1. Do you use specific outcome measure(s) to assess progress and treatment of your patients? [ 1 ] Yes [ 2 ] No
2. **If YES, State the outcome measure(s) you use e. g. Barthel Index, Berg balance scale etc**

**________________________________________________________________________**

**________________________________________________________________________**

**________________________________________________________________________**

**_________________________________________________________________________**

**SECTION D: REHABILITATION EQUIPMENT AVAILABLE AT FACILITY**

**Please, TICK (√)** the rehabilitation equipment available at your clinic (e.g. Pulley system, Vibro-massager, Parallel bar, Interferential therapy, Wax therapy etc).

| **SN** | **Name of Equipment** | **Available (Tick)** |
| --- | --- | --- |
| 1. | Wall bar |  |
| 2. | Parallel bar with mirror |  |
| 3. | Nautical wheel |  |
| 4. | Quadriceps bench |  |
| 5. | Tilt bed |  |
| 6. | Tread mill |  |
| 7. | Cycling ergonometer / Bike |  |
| 8. | Stair case |  |
| 9. | Dumb bells |  |
| 10. | Exercise balls & mats |  |
| 11. | Pulley system |  |
| 12. | Wax therapy |  |
| 13. | Cervical/lumbar traction |  |
| 14. | TENS machine |  |
| 15. | Short wave diathermy |  |
| 16. | Ultrasound therapy |  |
| 17. | Microwave diathermy |  |
| 18. | Infra-red/heat packer |  |
| 19. | Suspension cage/frame |  |
| 20. | Wobble board |  |
| 21 | Vibro-massager |  |
| 22 | Passive active trainer |  |
| 23 | Rods/cones |  |
| 24 | Play dough/beads |  |
| 25 | Hand dynamometer |  |
| 26 | Goniometer |  |
| 27 | Commode (Bed pan/chamber pot) |  |
| 28 | Walking frame |  |
| 29 | Wheelchairs |  |
| 30 | Electronic Pulse Massager |  |
| 31 | Blood Circulatory Machine |  |
| 32 | Ceragem Massaging Bed |  |
| 33 | Interferential therapy |  |
| 34 | **Other/please specify** |  |

**APPENDIX II PROCESS OF CARE**

**Instructions**

| **S/N** | **To what extent do you carry out these at your facility:** | **Very great extent**  **6** | **Great extent**  **5** | **moderate extent**  **4** | **small extent**  **3** | **Very small extent**  **2** | **Not at all**  **1** |
| --- | --- | --- | --- | --- | --- | --- | --- |
| 1 | Multidisciplinary team coordination | 6 | 5 | 4 | 3 | 2 | 1 |
| 2 | Baseline assessment of patients | 6 | 5 | 4 | 3 | 2 | 1 |
| 3 | Goal setting for patients | 6 | 5 | 4 | 3 | 2 | 1 |
| 4 | Planning of treatment | 6 | 5 | 4 | 3 | 2 | 1 |
| 5 | Monitoring and evaluating progress of patients | 6 | 5 | 4 | 3 | 2 | 1 |
| 6 | Management of impairments | 6 | 5 | 4 | 3 | 2 | 1 |
| 7 | Management of functional limitations | 6 | 5 | 4 | 3 | 2 | 1 |
| 8 | Prevention of complications | 6 | 5 | 4 | 3 | 2 | 1 |
| 9 | Prevention of a recurrent stroke | 6 | 5 | 4 | 3 | 2 | 1 |
| 10 | Patient & Family involvement in treatment goals | 6 | 5 | 4 | 3 | 2 | 1 |
| 11 | Patient education | 6 | 5 | 4 | 3 | 2 | 1 |
| 12 | Family education | 6 | 5 | 4 | 3 | 2 | 1 |
| 13 | Use of group circuit class therapy | 6 | 5 | 4 | 3 | 2 | 1 |
| 14 | Encouraging patients to continue therapy sessions at home. | 6 | 5 | 4 | 3 | 2 | 1 |
| 15 | Planning for discharge | 6 | 5 | 4 | 3 | 2 | 1 |

I will like to measure the process of care for stroke patients by compliance with the agency for healthcare policy and research (AHCPR) and Clinical guidelines for post-stroke rehabilitation.

**Please circle [ ] the number that best fits your response for each of the questions asked.**

**APPENDIX III**

**SECTION A: DEMOGRAPHIC INFORMATION OF PERSONS WITH STROKE**

1. Participant’s code: _______________
2. Date of completing this form (dd/mm/yyyy): _____________
3. Participant/Carer contact number: ________________________________
4. Address: ______________________
5. Age (years): ____________________
6. Gender: [ 1 ] Male [ 2 ] Female [ 3 ] other/please specify _________________
7. Occupation: ______________________________________
8. Marital status: [ 1 ] Single [ 2 ] Married [ 3 ] Divorced [ 4 ] Separated

[ 5 ] Cohabitating [ 6 ] Widow/er [ 7 ] Other (please specify) ______________

1. Level of Education: [ 1 ] None [ 2 ] Primary School [ 3 ] Junior High School

[4 ] Senior High School [ 5 ] Diploma/Degree [ 6 ] Postgraduate

1. Religion: [1] Christian [2]Moslem [3]Traditionalist

[4] Other/please specify______________________

**SECTION B: MEDICAL PROFILE**

1. Date of stroke occurrence (dd/mm/yyyy): _________________
2. Date of starting rehabilitation (dd/mm/yyyy): _______________
3. Number of days after the stroke (post stroke days): ___________ days
4. What diagnostic test(s) have you done already:

[1] CT scan [2] MRI [3] Both CT scan and MRI

[ 4 ] Other/please state _______________________________________

1. Side of body impairment: [ 1 ] Right [ 2 ] Left [ 3 ] Both
2. Type of stroke: [ 1 ] Ischaemic [ 2 ] Haemorrhagic [ 3 ] Undetermined
3. Were you admitted in the hospital when you first suffered the stroke? [ 1 ] Yes [ 2 ] No

8. If you answered **YES in question 7**, provide the number of hospitals days on admission: ___________ days

9. Who referred you to this facility? [ 1] self-referral [ 2 ] family member

[ 3 ] neighbour [ 4 ] advert on radio [ 5] hospital worker (e.g. doctor, nurse etc.)

10. How many times do you receive your rehabilitation treatment? [ 1 ] everyday

[2 ] once a week [ 3 ] twice a week [ 4 ] thrice a week [5] once a month

[6] Other/please state _______________________________________________________

11. What is the duration of your treatment session in a day?  **___________ minutes/hours.**

**SECTION C: REHABILITATION SERVICES**

1**.** Please tick (√) the rehabilitation services you have received for your condition:

[1] physiotherapy [2] occupational therapy [3] speech therapy [4] diet therapy

[5] psychotherapy [6] other/please specify________________________________

2. Please indicate the type, frequency and duration of rehabilitations services you have received:

| **SN** | **Type of rehabilitation e. g. physiotherapy** | **Frequency e. g. once a week** | **Duration in minutes** |
| --- | --- | --- | --- |
| 1 |  |  |  |
| 2 |  |  |  |
| 3 |  |  |  |
| 4 |  |  |  |
| 5 |  |  |  |
| 6 |  |  |  |
| 7 |  |  |  |
| 8 |  |  |  |
| 9 |  |  |  |
| 10 |  |  |  |

**SECTION D: SOCIO-ECONOMIC STATUS OF PATIENT**

1. Were you on paid employment when you had the stroke? [ 1 ] Yes [ 2 ] No

**If you answered NO in question 1, please proceed to question 4.**

1. Which of the following best describes your employment status before you had the stroke?

[ 1] Employed either in public or private sector

[2] Self-employed or helping in a family business

[3] Other/Please specify _________________________

1. Which of the following best describes your working hours? [1] Full time

[2] Part-time [3] Other/Please specify_________________________

1. What was the reason for not working?

[1] Unemployed, looking for work

[2] Unemployed, preferred not to work

[3] Unable to work due to illness or disability (receiving payment from my employers)

[4] Unable to work due to illness or disability (not receiving any pay)

[5] Retired/pensioner

[6] Looking after the home, no income/benefits (including looking after children)

[7] Student

[8] Other/ Please specify_____________________________________

1. What is/was your monthly income? If this income varies from one month to another, please give an average.

[ 1 ] Less than 200 cedis

[ 2 ] 201 to 500 cedis

[ 3 ] 501 to 1000 cedis

[ 4 ] 1001 to 2000 cedis

[ 5 ] 2001 to 3000 cedis

[ 6 ] More than 3000 cedis

1. Can you make ends meet with your total monthly disposable income?

[ 1 ] Yes [ 2 ] No

1. If you answered **No in question 6**, how do you make ends meet? _______________________________________________________________________

**Thank you.**

**APPENDIX IV**

**QUALITATIVE INTERVIEW GUIDE**

**Section A: General information**

1. Participant’s code: _______________
2. Contact number: _________________ Alternative contact: _____________________
3. Date of interview (dd/mm/yyyy): ___________________
4. Time of interview: Start ____________ End: ________________
5. Place of interview: ________________
6. Address of interviewee: ______________________
7. Age of interviewee : ____________________
8. Gender of interviewee : _________________
9. Marital Status: a) Single b) Married c) Divorced

d) Widowed e) other/specify____________________

1. Side of affection: a) Right b) Left
2. Level of Education: a) None b) Primary School c) Secondary School d) Tertiary
3. Occupation: ___________________________
4. Religion: a) Christian b) Moslem c) Traditionalist

d)other/please specify _____________________

**Guide**

1. Please tell me about how your illness started?
2. Describe to me all the care you have received since you had the stroke?

**Probe: what happened at the hospital, how long did it take for you to be attended to? What**

**treatment(s) was/were given to you? What care did you receive and how was it?**

1. How will you describe the professionals attitude, knowledge and skills about your condition?
2. How will you describe the interaction/relationship between you and professionals concerning

your care?

**Probe: how did they involve you in the care process, how did they involve you in the treatment?**

**How was your family involved in the care?**

1. To what extent did the professionals provide you with information about your condition and

progress of therapy?

1. What were/are your experiences about the care you receive?

**Probe: How do you feel during the process of receiving care at the facility?**

1. What are your experiences with how the care is organized here?

**Probe: your experiences about the professionals, rehabilitation care and your recovery?**

1. What are the challenges and facilitators you experienced with your care at home and in the facility?
2. Why will you recommend this facility/rehabilitation pathway to someone for stroke care?
3. What are the changes or modifications you think should be made to this place/facility?

**Thank you for your time and the information provided.**

**Any other thing you want to share with me?**

**APPENDIX V**

**MODIFIED RAKIN SCORE**

1. No symptoms at all
2. No significant disability despite symptoms; able to carry out all usual duties and activities
3. Slight disability; unable to carry out all previous activities, but able to look after own affairs without assistance
4. Moderate disability; requiring some help, but able to walk without assistance
5. Moderately severe disability; unable to walk without assistance and unable to attend to own bodily needs without assistance
6. Severe disability; bedridden, incontinent and requiring constant nursing care and attention
7. Dead

**TOTAL (0–6): _______**

**Barthel ADL Index**

**Instruction:**

Measure of physical disability used widely to assess behaviour relating to activities of daily living for stroke patients or patients with other disabling conditions. It measures what patients do in practice. Assessment is made by anyone who knows the patient well.

| **Item** | **Interpretation** | **Score** |
| --- | --- | --- |
| Bowel | Incontinent or needs enemas  Occasional accident (1X/week)  Continent | 0  5  10 |
| Bladder | Incontinent or needs enemas  Occasional accident (1X/week)  Continent | 0  5  10 |
| Grooming | Needs help with personal care  Independent (including face, hair, teeth, shaving, implements provided) | 0  5 |
| Toilet use | Dependent  Needs some help, but can do something alone  Independent (on and off, dressing and wiping) | 0  5  10 |
| Feeding | Unable  Needs help, e.g. cutting, spreading butter etc  Independent | 0  5  10 |
| Transfer (bed to chair and back) | Unable, no sitting balance  Major help (1 or 2 people, physical), can sit  Minor help (verbal or physical)  Independent | 0  5  10  15 |
| Mobility | Immobile  Wheelchair independent (including corners)  Walks with the help of 1 person (physical or  verbal help)  Independent (but may any use aid eg stick) | 0  5  10  15 |
| Dressing | Dependent  Needs help – can do half unaided  Independent (including buttons, zips, laces) | 0  5  10 |
| Stairs | Unable  Needs help (verbal or physical, carrying aid)  Independent | 0  5  10 |
| Bathing | Dependent  Independent (bath or shower) | 0  5 |
| **Total scoring** | |  |

**Interpretation of scoring**

| **SCORE** | **INTERPRETATION** |
| --- | --- |
| 00 - 20 | Total Dependence |
| 21 - 60 | Severe Dependence |
| 61 - 90 | Moderate Dependence |
| 91 - 99 | Slight Dependence |
| - 100 | Independence |

**APPENDIX VI**

**Stroke Rehabilitation Assessment of Movement (S T R E A M)**

PATIENT ID: ________ Date of Assessment: ____________ Facility: __________________

Date of next assessment: __________________________________________________

**INSTRUCTIONS**

**I. VOLUNTARY MOVEMENT OF THE LIMBS**

| **0** =unable to perform the test movement through any appreciable range (includes flicker or slight movement)  **1 a =** able to perform only part of the movement, and with marked deviation from normal pattern  **1 b** = able to perform only part of the movement, but in a manner that is comparable to the unaffected  side  **1 c=** able to complete the movement, but only with marked deviation from normal pattern  **2**= able to complete the movement in a manner that is comparable to the unaffected side  **X** = activity non tested (specify why; ROM, Pain, Other (reasons)) |
| --- |

**II. BASIC MOBILITY**

| **0** = unable to perform the test activity through any appreciable range (ie, minimal active participation)  **1 a =** able to perform only part of the activity independently (requires partial assistance or stabilization to  complete), with or without an aid, and with marked deviation from normal pattern  **1 b** = able to perform only part of the activity independently (requires partial assistance or stabilization to  complete), with or without an aid, but with a grossly normal movement pattern  **1 c** = able to complete the activity independently, with or without an aid, but only with marked deviation  from normal pattern  **2** = able to complete the activity independently with a grossly normal movement pattern, but requires an  aid  **3** = able to complete the activity independently with a grossly normal movement pattern, without an aid  **X** = activity non tested (specify why; ROM, Pain, Other (reason)) |
| --- |

| **SN** | **Domain/Item** | **score** |
| --- | --- | --- |
| **SUPINE** | | |
| 1. | Protracts scapula un supine  “Lift your shoulder blade so that your hand moves  towards the ceiling“  **Note: therapist stabilizes arm with shoulder 90° flexed**  **and elbow extended.** | /2 |
| 2. | Extends elbow in supine (starting with elbow fully  flexed)  “Lift your hand towards the ceiling, straightening your  elbow as much as you can“  **Note: therapist stabilizes arm with shoulder 90° flexed;**  **strong associated shoulder extension and/or**  **abduction= marked deviation (score la or lc).** | /2 |
| 3. | Flexes hip and knee in supine (attains half crook lying)  “Bend your hip and knee so that your foot rests flat on the  bed” | /2 |
| 4. | Rolls onto side (starting from supine)  “Roll onto your side”  **Note: may roll onto either side; pulling with arms to turn**  **over= aid (score 2).** | /3 |
| 5. | Raises hips off bed in crook lying (bridging)  “Lift your hips as high as you can”  **Note: therapist may stabilize foot, but if knee pushes**  **strongly into extension with bridging= marked deviation**  **(score 1a or 1c); if requires aid (external or from patient)** | /3 |
| 6. | Moves from lying supine to sitting (with feet on the  floor)  “Sit up and place your feet on the floor”  **Note: may sit up to either side using any functional and safe method; longer than 20 seconds= marked deviation**  **(score 1a or 1c); pulling up using bedrail or edge of plinth= aid (score 2).** | /3 |
| **SITTING (feet supported; hands resting on pillow on lap for items 7-14)** | | |
| 7. | Shrugs shoulders (scapular elevation)  “Shrug your shoulders as high as you can“  **Note: both shoulders are shrugged simultaneously.** | /2 |
| 8. | Raises hand to touch top of the head  “Raise your hand to touch the top of your head” | /2 |
| 9. | Places hand on sacrum  “Reach behind your back and as far across toward the  other side as you can” | /2 |
| 10. | Raises arm overhead to fullest elevation  “Reach your hand as high as you can towards the ceiling” | /2 |
| 11. | Supinates and pronates forearm (elbow flexed at 90°)  “Keeping your elbow bent and close to your side, turn  your forearm over so that your palm faces up, then turn  your forearm over so that your palm faces down”  **Note: movement in one direction only = partial movement**  **(score 1a or 1b).** | /2 |
| 12. | Closes hand from fully opened position  “Make a fist, keeping your thumb on the outside”  **Note: must extend wrist slightly (ie, wrist cocked) to obtain**  **full marks; full fist with lack of wrist extension = partial**  **movement (score 1a or 1b).** | /2 |
| 13. | Opens hand from fully closed position  “Now open your hand all the way” | /2 |
| 14. | Opposes thumb to index finger (tip to tip)  “Make a circle with your thumb and index finger” | /2 |
| 15. | Flexes hip in sitting  ”Lift your knee as high as you can“ | /2 |
| 16. | Extends knee in sitting  ”Straighten your knee by lifting your foot up“ | /2 |
| 17. | Flexes knee in sitting  “Slide your foot back under you as far as you can“  **Note: start with affected foot forward (heel in line with toes**  **of other foot).** | /2 |
| 18. | Dorsiflexes ankle in sitting  “Keep your heel on the ground and lift your toes off the  floor as far as you can”  **Note: affected foot is placed slightly forward (heel in line**  **with toes of other foot).** | /2 |
| 19. | Plantar flexes ankle in sitting  “Keep your toes on the ground and lift your heel off the  floor as far as you can” | /2 |
| 20. | Extends knee and dorsiflexes ankle in sitting  “Straighten your knee and bring your toes towards you”  **Note: extension of knee without dorsiflexion of ankle=**  **partial movement (score 1a or 1b).** | /2 |
| 21. | Rises to standing from sitting  “Stand up; try to take equal weight on both legs”  **Note: pushing up with hand(s) to stand= aid (score 2);**  **asymmetry such as trunk lean, Trendelenburg position,**  **hip retraction, or excessive flexion or extension of the**  **affected knee= marked deviation (score 1a or 1c).** | /3 |
| **STANDING** | | |
| 22. | Maintains standing for 20 counts  “Stand on the spot while I count to twenty” | /3 |
| **STANDING (holding onto a stable support to assist balance for items 23-25)** | | |
| 23. | Abducts affected hip with knee extended  “Keep your knee straight and your hips level, and raise  your leg to the side” | /2 |
| 24. | Flexes affected knee with hip extended  “Keep your hip straight, bend your knee back and bring  your heel towards your bottom” | /2 |
| 25. | Dorsiflexes affected ankle with knee extended  “Keep your heel on the ground and lift your toes off the  floor as far as you can”  **Note: affected foot is placed slightly forward in position of**  **a small step (heel in line with toes of other foot).** | /2 |
| **STANDING AND WALKING ACTIVITES** | | |
| 26. | Places affected foot onto first step (or stool 18 cm  high)  “Lift your foot and place it onto the first step (or stool) in  front of you”  **Note: returning the foot to the ground is not scored; use of**  **handrail= aid (score 2).** | /3 |
| 27. | Takes 3 steps backwards (one and a half gait cycles)  “Take three average sized steps backwards, placing one  foot behind the other” | /3 |
| 28. | Takes 3 steps sideways to affected side  “Take three average sized steps sideways towards your  weak side” | /3 |
| 29. | Walks 10 meters indoors (on smooth, obstacle= free  surface)  “Walk in a straight line over to … (a specified point 10  meters away)”  **Note: orthotic= aid (score 2); longer than 20 seconds=**  **marked deviation (score 1c).** | /3 |
| 30. | Walks down 3 stairs alternating feet  “Walk down three stairs; place only one foot at a time on  each step if you can”  **Note: handrail= aid (score 2); non-alternating feet=**  **marked deviation (score 1a or 1c).** | /3 |
| **TOTAL SCORES** | |  |

**MOVEMENT QUALITY**

|  | **None** | **Partial** | **Complete** |
| --- | --- | --- | --- |
| Marked Deviation | 0 | 1 a | 1 c |
| Grossly normal | 0 | 1 b | 2 (3) |


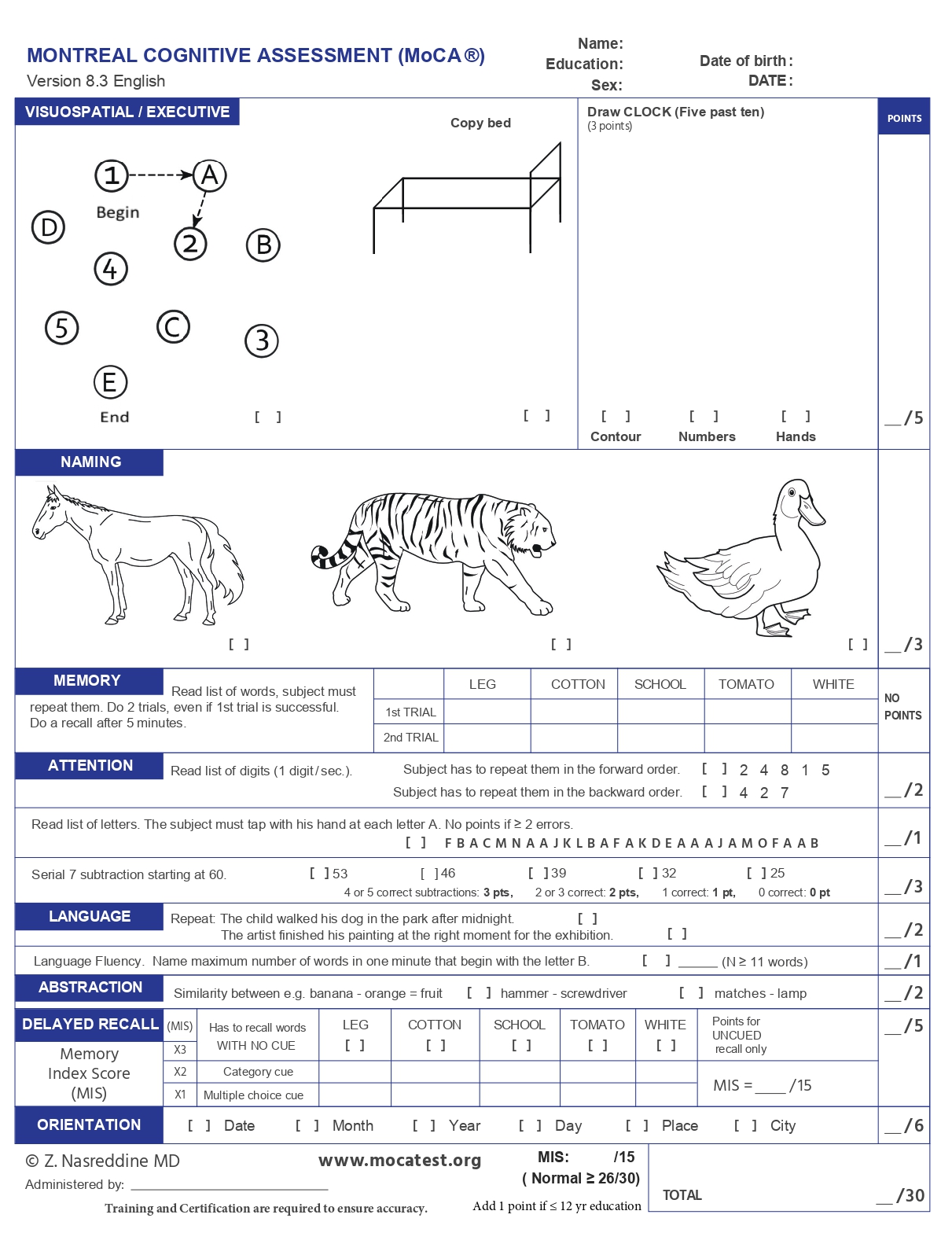


**APPENDIX VIII**

**Timed 10-Meter Walk Test**

PATIENTS ID: ________Facility: ___________ Date of Assessment: _____________

Date of next assessment: _________________________________________________

**INSTRUCTION:**

The individual walks without assistance 10 meters (32.8 feet) and the time is measured for the intermediate

6 meters (19.7 feet) to allow for acceleration and deceleration.

- start timing when the toes of the leading foot cross the 2-meter mark
- stop timing when the toes of the leading foot cross the 8-meter mark
- assistive devices can be used but should be kept consistent and documented from test to test
- if physical assistance is required to walk; assessment can be performed at preferred walking speed

or fastest speed possible.

- documentation should include the speed tested (preferred vs. fast)
- collect three trials and calculate the average of the three trials

**SET-UP**

- Measure and mark a 10-meter walkway
- Add a mark at 2-meters
- Add a mark at 8-meters

**Patient Instructions:**

*Normal comfortable speed*: “I will say ready, set, go. When I say go, walk at your normal comfortable speed

until I say stop”

*Maximum speed trials:* “I will say ready, set, go. When I say go, walk as fast as you safely can until I say stop”

**RECORDING:**

Seconds to ambulate 10 meters (only the middle 6 meters are timed)

Self-Selected Velocity: Trial 1________sec. Fast Velocity: Trial 1________sec.

Self-Selected Velocity: Trial 2_______sec. Fast Velocity: Trial 2________sec.

Self-Selected Velocity: Trial 3 _______sec. Fast Velocity: Trial 3________sec.

**Self-Selected Velocity: Average time________sec. Fast Velocity: Average time_________sec.**

Actual velocity: Divide 6 metres by the average time in seconds

**Average Self-Selected Velocity: _________m/s**

**Average Fast-Velocity: ________________m/s**

**APPENDIX IX**

**TIMED UP AND GO (TUG) TEST**

PATIENTS ID: ________Facility: ___________ Date of Assessment: _____________

Date of next assessment: _________________________________________________

**Equipment:** Stopwatch

Standard Chair with arm rest

Measured distance of 3 meters (10feet)

**Patient Instructions:**

“My commands for this test are going to be ‘ready, set, go’. When I say go, I want you to stand up from the chair. You may use the arms of the chair to stand up or sit down. Once you are up, you may take any path you like, but I want you to move as QUICKLY as you feel safe and comfortable until you pass this piece of tape (or end of marked course) with both feet. Turn around and walk back to the chair. I will stop the clock when your back touches the back of the chair. **You will complete one practice run and two that are counted.”**

The subject is allowed to use an assistive device. Be sure to document the assistive device used.

**Therapist Instructions:** Start timing on the word “GO” and stop timing when the subject is seated again correctly in the chair with their back resting on the back of the chair. The subject wears their regular foot wear, may use any gait aid that they normally use during ambulation, but may not be assisted by another person. There is no time limit. They may stop and rest (but not sit down) if they need to.

**NB: A practice trial should be completed before the timed trial**

**RECORDING:** Time in seconds

Time 1: ________________________________________________

Time 2: ________________________________________________

Time 3: ________________________________________________

**APPENDIX X**

**TINETTI BALANCE ASSESSMENT TOOL**

PATIENTS ID: ________Facility: ___________ Date of Assessment: _____________

Date of next assessment: _________________________________________________

**BALANCE SECTION**

**Patient is seated in hard, armless chair**

| **Item** | **Activity** | **score** |
| --- | --- | --- |
| Sitting Balance | Leans or slides in chair  Steady, safe | 0  1 |
| Rises from chair | Unable to without help  Able, uses arms to help  Able without use of arms | 0  1  2 |
| Attempts to rise | Unable to without help  Able, requires > 1  Able to rise, 1 attempt | 0  1  2 |
| Immediate standing balance (first 5 seconds) | Unsteady (staggers, moves feet, trunk sway)  Steady but uses walker or other support  Steady without walker or other support | 0  1  2 |
| Standing balance | Unsteady  Steady but wide stance and uses support  Narrow stance without support | 0  1  2 |
| Nudged | Begins to fall  Staggers, grabs, catches self  Steady | 0  1  2 |
| Eyes closed | Unsteady  Steady | 0  1 |
|  | Discontinuous steps  Continuous | 0  1 |
| Turning 360 degrees | Unsteady (grabs, staggers)  Steady | 0  1 |
| Sitting down | Unsafe (misjudged distance, falls into chair)  Uses arms or not a smooth motion  Safe, smooth motion | 0  1  2 |
| **Total Balance score** |  | **/16** |

**GAIT SECTION**

**Patient stands with therapist, walks across room (+/- aids), first at usual pace, then at rapid pace.**

| **Item** | **Activity** | **Score** |
| --- | --- | --- |
| Indication of gait  (Immediately after told to ‘’go’’) | Any hesitancy or multiple attempts  No hesitancy | 0  1 |
| Step length & height | RIGHT swing food does not pass left stance foot with step  RIGHT foot passes left stance foot  RIGHT foot does not clear floor completely with step  RIGHT foot completely clears floor | 0  1  0  1 |
| Step length & height | LEFT swing foot does not pass right Stance foot with step  LEFT foot passes right stance foot  LEFT foot does not clear floor completely with step  LEFT foot completely clears floor | 0  1  0  1 |
| Foot clearance | Foot drop  L foot clears floor  R foot clears floor | 0  1  1 |
| Step symmetry | Right and left step length not equal  Right and left step length appear equal | 0  1 |
| Step continuity | Stopping or discontinuity between steps  Steps appear continuous | 0  1 |
| Path | Marked deviation  Mild/moderate deviation or uses walking aid  Straight without walking aid | 0  1  1 |
| Trunk | Marked sway or uses walking aid  No sway but flex knees or back or uses arms for stability  No sway, flexion, use of arms or walking aid | 0  1  2 |
| Walking time | Heel apart  Heels almost touching while walking | 0  1 |
| **Total gait score** | | **/12** |
|  |  |  |
| **Grand score = balance + gait score** | |  |

**APPENDIX XI**

**HEALTH RELATED QUALITY OF LIFE FOR STROKE PATIENTS**

PATIENTS ID: ________Facility: ___________ Date of Assessment: _____________

Date of next assessment: _________________________________________________

**Instructions**

This assessment asks about how you perceive your current state of health, quality of life, or other areas of your life. **Please answer all the questions honestly as they put into categories**. If you are unsure about what response to give to a question, please choose the nearest most appropriate response.

**Please keep in mind your standards, hopes, pleasures and concerns. Think about your life in the last two weeks. Kindly circle** [ ] **the number that best fits your response.**

| **PHYSICAL DOMAIN** | | **Not at all**  **(1)** | **A little**  **(2)** | **A moderate amount**  **(3)** | **Very much**  **(4)** | **Extremely**  **(5)** |
| --- | --- | --- | --- | --- | --- | --- |
| 1 | To what extent do you have difficulties gripping objects, turning door-knob, using cutlery, writing, opening jar/can, carrying heavy objects? | 1 | 2 | 3 | 4 | 5 |
| 2 | To what extent do you have difficulties sitting/standing without losing your balance? | 1 | 2 | 3 | 4 | 5 |
| 3 | To what extent do you think physical pain/discomfort /abnormal sensation/absent sensation prevent you from doing what you need to? | 1 | 2 | 3 | 4 | 5 |
|  |  | **Very dissatisfied (1)** | **Dissatisfied**  **(2)** | **Neither satisfied nor dissatisfied(3)** | **Satisfied**  **(4)** | **Very satisfied**  **(5)** |
| 4 | How satisfied are you with your ability to perform your daily living activities [feeding, bathing, toileting, dressing, grooming, e.t.c.]? | 1 | 2 | 3 | 4 | 5 |
| 5 | How satisfied are you with your capacity for work? | 1 | 2 | 3 | 4 | 5 |
| 6 | How satisfied are you with your sex life? | 1 | 2 | 3 | 4 | 5 |
| 7 | **How important to you are the aspects of your life covered in the physical domain?** | **Not at all**  **(1)** | **A little**  **(2)** | **A moderate amount (3)** | **Very much (4)** | **Extremely**  **(5)** |
| **EMOTION/PSYCHOLOGICAL DOMAIN** | | **Not at all**  **(1)** | **A little**  **(2)** | **Moderately**  **(3)** | **Very much**  **(4)** | **Always** **(5)** |
| 1 | How often do you have negative feelings such as blue mood, anger, despair, anxiety, depression, fear? | 1 | 2 | 3 | 4 | 5 |
| **2** | Do you have enough energy for everyday life? | 1 | 2 | 3 | 4 | 5 |
| **3** | To what extent are you able to accept your bodily appearance? | 1 | 2 | 3 | 4 | 5 |
| **4** | To what extent do you enjoy your work? | 1 | 2 | 3 | 4 | 5 |
| **5** | How often do you laugh? | 1 | 2 | 3 | 4 | 5 |
| **6** | To what extent do you enjoy recreation/pastimes/leisure/rest/ relaxation | 1 | 2 | 3 | 4 | 5 |
| **7** | How satisfied are you with your feelings? | 1 | 2 | 3 | 4 | 5 |
| **8** | **How important to you are the aspects of your life covered in the psychological domain?** | 1 | 2 | 3 | 4 | 5 |

| **INTELLECTUAL/COGNITIVE DOMAIN** | | | **Not at all**  **(1)** | **A little**  **(2)** | **Moderately**  **(3)** | **Very much**  **(4)** | **Extremely**  **(5)** |
| --- | --- | --- | --- | --- | --- | --- | --- |
| **1** | | How well are you able to concentrate? | 1 | 2 | 3 | 4 | 5 |
| **2** | | How available to you is the information that you need for your day-to-day life? | 1 | 2 | 3 | 4 | 5 |
| **3** | | To what extent are you able to communicate? | 1 | 2 | 3 | 4 | 5 |
|  | |  | **Very dissatisfied (1)** | **Dissatisfied**  **(2)** | **Neither satisfied nor dissatisfied(3)** | **Satisfied**  **(4)** | **Very satisfied**  **(5)** |
| **4** | | How satisfied are you with your ability to communicate? | 1 | 2 | 3 | 4 | 5 |
| **5** | | How satisfied are you with your ability to think and learn? | 1 | 2 | 3 | 4 | 5 |
| **6** | | **How important to you are the aspects of your life covered in the intellectual or cognitive domain?** | **Not at all**  **(1)** | **A little**  **(2)** | **Moderately**  **(3)** | **Very much**  **(4)** | **Extremely**  **(5)** |
| **SOUL DOMAIN** | | | **Not at all**  (1) | **Little**  **(2)** | **Moderate**  **(3)** | **Very Much**  **(4)** | **Extremely**  **(5)** |
| **1** | How much confidence do you have in yourself? | | 1 | 2 | 3 | 4 | 5 |
| **2** | To what extent do you believe you have a purpose for living? | | 1 | 2 | 3 | 4 | 5 |
| **3** | To what extent are you interested in fulfilling your purpose for living? | | 1 | 2 | 3 | 4 | 5 |
| **4** | To what extent do you practice your religion/faith? | | 1 | 2 | 3 | 4 | 5 |
|  |  | | **Very dissatisfied**  **(1)** | **Dissatisfied**  **(2)** | **Neither satisfied nor dissatisfied (3)** | **Satisfied**  **(4)** | **Very satisfied**  **(5)** |
| **5** | To what extent are you satisfied with your faith in God? | | **1** | **2** | **3** | **4** | **5** |
| **6** | How satisfied are you with yourself? | | **1** | **2** | **3** | **4** | **5** |
| **7** | **How important to you are the aspects of your life covered in the soul domain?** | | **Not at all**  **(1)** | **A little**  **(2)** | **Moderately**  **(3)** | **Very much (4)** | **Extremely**  **(5)** |
| **SPIRITUAL DOMAIN** | | | **Not at all**  **(1)** | **A little**  **(2)** | **Moderately**  **(3)** | **Very**  **much**  **(4)** | **Extremely**  **(5)** |
| **1** | To what extent do you understand God? | | 1 | 2 | 3 | 4 | 5 |
| **2** | To what extent are you guided / motivated by God in your daily life? | | 1 | 2 | 3 | 4 | 5 |
| **3** | To what extent do you understand your religion/faith? | | 1 | 2 | 3 | 4 | 5 |
|  |  | | **Very**  **Dissatisfied (1)** | **Dissatisfied (2)** | **Neither satisfied nor dissatisfied(3)** | **Satisfied**  **(4)** | **Very satisfied**  **(5)** |
| **4** | To what extent are you satisfied with divine guidance in your life? | | 1 | 2 | 3 | 4 | 5 |
| **5** | **How important to you are the aspects of your life covered in the spiritual and soul domain?** | | **Not at all**  **(1)** | **A little**  **(2)** | **Moderately**  **(3)** | **Very much**  **(4)** | **Extremely**  **(5)** |
| **SPIRITUAL INTERACTION DOMAIN** | | | **Not at all**  (1) | **Little**  **(2)** | **Moderate**  **(3)** | **Very Much**  **(4)** | **Extremely**  **(5)** |
| 1 | To what extent do you consider yourself close to God or your object of worship? | | 1 | 2 | 3 | 4 | 5 |
| **2** | To what extent do you discuss aspects of your faith/religion with people of the same religious interest/faith in order to strengthen your individual resolve? | | 1 | 2 | 3 | 4 | 5 |
|  |  | | **Very dissatisfied**  (1) | **Dissatisfied**  (2) | **Neither satisfied nor dissatisfied(3)** | **Satisfied**  **(4)** | **Very satisfied**  **(5)** |
| **3** | How satisfied are you with your relationship with God or your object of worship? | | 1 | 2 | 3 | 4 | 5 |
| **4** | How satisfied are you with your effort to develop your faith/religion? | | 1 | 2 | 3 | 4 | 5 |
| **5** | **How important to you are the aspects of your life covered in the spiritual interaction domain?** | | **Not at all (1)** | **A little (2)** | **Moderately (3)** | **Very much (4)** | **Extremely**  **(5)** |
| **ECONOMIC/ SOCIAL DOMAIN** | | | **Fully dependent (1)** | **Requires substantial help (2)** | **Requires minimal help (3)** | **Requires no help but not back to work (4)** | **Back to work**  **(5)** |
| **1** | Performing activities of daily living e.g. feeding, bathing, toileting etc. | | 1 | 2 | 3 | 4 | 5 |
|  |  | | **Not at all (1)** | **A little (2)** | **Moderately (3)** | **Very much (4)** | **Completely (5)** |
| **2** | How much respect do you get from others? | | 1 | 2 | 3 | 4 | 5 |
| **3** | How well are you able to manage your home and perform your domestic roles? | | 1 | 2 | 3 | 4 | 5 |
| **4** | To what extent do you have access to transport facilities? | | 1 | 2 | 3 | 4 | 5 |
|  |  | | **Very dissatisfied**  (1) | **Dissatisfied**  **(2)** | **Neither satisfied nor dissatisfied (3)** | **Satisfied (4)** | **Very satisfied**  **(5)** |
| **5** | How satisfied are you with your personal relationships? | | 1 | 2 | 3 | 4 | 5 |
| **6** | How satisfied are you with the support you get from your friends? | | 1 | 2 | 3 | 4 | 5 |
| **7** | How satisfied are you with your access to health services? | | 1 | 2 | 3 | 4 | 5 |
| **8** | **How important to you are the aspects of your life covered in the economic/social domain?** | | **Not at all**  **(1)** | **A little**  **(2)** | **Moderately**  **(3)** | **Very much**  **(4)** | **Extremely**  **(5)** |

**Thank you for your time**

**APPENDIX XII**

**THE REINTEGRATION TO NORMAL LIVING INDEX (RNLI)**

**INSTRUCTION:** Scoring is based on distance along a 10 cm visual analogue scale (VAS).

Indicate where wheelchairs or other adaptive aids may be used.

| **Response** | **Score** |
| --- | --- |
| No integration | 0 |
| Complete integration | 10 |

Total score = SUM (points for all 11 items)

Adjusted score = (total score) / 110 * 100

**SCORING:**

**QUESTIONS:**

(1) I move around my living quarters as I feel necessary. ___________________

(2) I move around my community as I feel necessary. _____________________

(3) I am able to take trips out of town as I feel are necessary. _______________

(4) I am comfortable with how my self-care needs (dressing feeding toileting bathing) are met. _____________

(5) I spend most of my days occupied in work activity that is necessary or important to me. ________________

(6) I am able to participate in recreational activities (hobbies crafts sports reading television games computers etc.) as I want to. ____________________

(7) I participate in social activities with family, friends and or business acquaintances as is necessary or desirable to me. _____________________

(8) I assume a role in my family which meets my needs and those of other family members. ________________

(9) In general, I am comfortable with my personal relationships. ______________________

(10) In general, I am comfortable with myself when I am in the company of others. ________

(11) I feel that I can deal with life events as they happen. ________________________

**Thank you**

**STROKE LEVITY SCALE**

The SLS is a 4-item scale that evaluates stroke severity in terms of upper extremity and lower extremity motor performance, presence or absence of aphasia and mobility. The scale takes approximately two minutes to administer

| I | **Best motor power in the dexterous hand/upper limb** | 0  nil | 1 flicker | 2 gravity eliminated | 3 against gravity | 4 against resistance | 5 normal |
| --- | --- | --- | --- | --- | --- | --- | --- |
| Ii | **Best motor power in the affected upper limb** | 0 | 1 | 2 | 3 | 4 | 5 |
| iii | **Best motor power in the affected lower limb** | 0 | 1 | 2 | 3 | 4 | 5 |
| iv | **Speech defect/aphasia** | 0 nil | | | 1 present | | |

**INTERPRETATION:**

The total obtainable score on the SLS is 15 and lower score denotes higher stroke severity.

**Severe stroke** - Score of 0–5

**Moderate severity** – Score of 6–10

**Mild severity** – Scores of 11–15
